# An adult case of lymphangioma of the hepatoduodenal ligament mimicking a hepatic cyst

**DOI:** 10.1186/s40792-016-0280-0

**Published:** 2017-01-03

**Authors:** Toshihiko Kohashi, Toshiyuki Itamoto, Yasuhiro Matsugu, Takashi Nishisaka, Hideki Nakahara

**Affiliations:** 1 Department of Gastroenterological Surgery, Hiroshima Prefectural Hospital, 1-5-54 Ujinakanda, Minami-ku, Hiroshima 734-8530 Japan; 2 Department of Gastroenterological and Transplant Surgery, Applied Life Sciences, Institute of Biomedical and Health Sciences, Hiroshima University, 1-2-3 Kasumi, Minami-ku, Hiroshima 734-8551 Japan; 3 Department of Pathology, Hiroshima Prefectural Hospital, 1-5-54 Ujinakanda, Minami-ku, Hiroshima 734-8530 Japan

**Keywords:** Lymphangioma, Liver cyst, Hepatoduodenal ligament

## Abstract

**Background:**

Intra-abdominal lymphangiomas are rare, benign tumors in adults. This report is the third documented case of a lymphangioma originating in the hepatoduodenal ligament that mimicked a simple liver cyst.

**Case presentation:**

A 50-year-old woman was admitted with a cystic tumor in the right lobe of her liver and underwent laparoscopic excision of the cyst. Operative findings revealed that the cyst had developed in the hepatoduodenal ligament, not in the liver. A small part of the cystic wall remained on the dorsal surface of the hepatoduodenal ligament. Immunohistochemically, the tumor cells stained positive only for D2-40, leading to a diagnosis of lymphangioma (cystic type). Six months later, a cystic tumor recurred and was completely excised via laparotomy. No recurrence was observed after the second operation.

**Conclusions:**

The incomplete excision of the cystic tumor led us to re-operate 6 months after the first operation for the local disease recurrence.

## Background

Lymphangiomas are rare, benign tumors and most of them occur in children. Approximately 90% of these childhood patients are diagnosed within the second year of life. More than 90% of lymphangiomas are located in extra-abdominal regions, such as the head, neck, and axilla. Lymphangiomas in the peritoneal cavity are extremely rare, particularly in adults [1–4]. Herein, we describe a rare adult case of lymphangioma arising from the hepatoduodenal ligament, mimicking a simple liver cyst.

## Case presentation

A 50-year-old woman who had been treated 2 years ago with hormone preparations administered tamoxifen and leuprorelin acetate after a curative operation for solid-tubular carcinoma in the left breast was hospitalized with a 4-month history of abdominal distension and epigastric discomfort. On physical examination, there was slight tenderness in her epigastric region with normal bowel sounds. Laboratory examinations were unremarkable.

**Fig. 1:**
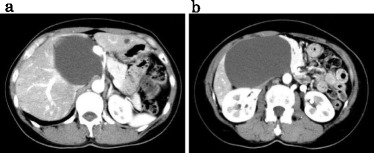
**a**, **b** Computed tomography images showing a large cystic mass between the hilar region and anterior segment of the liver without septa

An ultrasound sonography (US) revealed a low echoic, large cystic mass in the epigastrium without a septum in the tumor. Computed tomography (CT) scans with contrast medium demonstrated a low-density, homogeneous tumor without a septum. The tumor was oval in shape, 13 × 10 cm in diameter, and attached to the inferior surface of the liver. The cystic wall was not enhanced by the contrast medium (Fig. 1). These findings suggested a unilocular cyst of the liver.

**Fig. 2:**
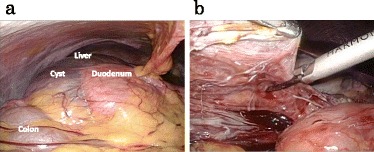
Laparoscopy image showing a large cyst at the caudal portion of the right lobe of the liver, which is compressing the duodenum to the left and the transverse colon downward (**a**). The cyst wall was resected using an ultrasonic coagulation and cutting device (**b**)

Laparoscopy revealed a large cyst at the caudal site of the right lobe of the liver that compressed the duodenum to the left and the transverse colon downward (Fig. 2a). Under the diagnosis of simple liver cyst, the cyst was mobilized from the duodenum, transverse colon, and omentum. However, during the operative procedure, we noticed that the cyst originated from the hepatoduodenal ligament. After aspiration of the contents in the cyst that was serous fluid with slightly turbid, the cyst wall was excised as extensively as possible (Fig. 2b). Finally, we cauterized the inner layer of the residual cystic wall with bipolar forceps.

**Fig. 3:**
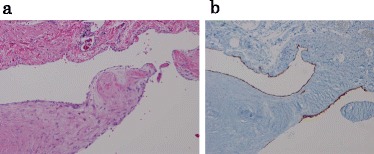
The hematoxylin-eosin-stained specimen revealing flattened endothelium lining the inner-surface of the wall (**a**) (×100). The endothelium that lined the inner-surface of the cystic tumor wall stained positive for D2-40 (**b**) (×100)

Hematoxylin-eosin staining revealed that the cystic wall was fibrotic with collagen fiber and flattened endothelium lining the inner-surface (Fig. 3a). Immunohistochemically, the flattened endothelium was negative for cytokeratin, calretinin, and HBME-1, markers of mesothelium. The endothelium was also negative for CD-34, a marker of vascular endothelial cells, but was positive for D2-40, indicating a composition of lymphatic endothelial cells (Fig. 3b). Considering the histological and immunohistochemical findings, we made the diagnosis of lymphangioma originating from the hepatoduodenal ligament. Postoperative treatment went well, and the patient was subsequently discharged.

**Fig. 4:**
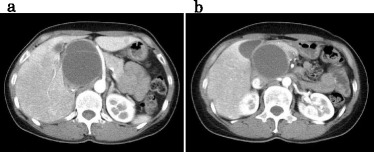
**a**, **b** Computed tomography images showing a recurrent cystic mass with septa in the same region of the first operation

**Fig. 5:**
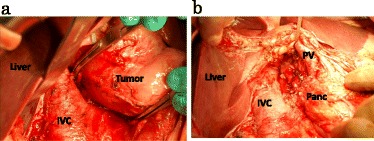
The recurrent cyst adhered to the inferior vena cava, common bile duct, and portal vein (**a**). It was excised completely via laparotomy (**b**)

Six months later, the patient re-presented with abdominal distension and epigastric discomfort, and CT revealed a recurrent cyst in the same region of the previous resection (Fig. 4a, b). The recurrent cyst was excised completely via laparotomy, which was performed because of a dense adhesion between the cyst and the inferior vena cava, common bile duct, and portal vein (Fig. 5a, b). The patient was alive without recurrence 1 year and 6 months after the second operation.

### Discussion

Intra-abdominal lymphangiomas are rare, and 70% of these tumors are found in the mesentery of the small or large bowel. There are a few reports of such tumors in the retroperitoneum, omentum, gastrointestinal tract, liver, pancreas, or spleen [5]. Only two other reports describing a lymphangioma in the hepatoduodenal ligament had been published previously (Table 1) [6, 7].

**Table 1:** Characteristics of adult cases of lymphangioma originated from hepatoduodenal ligament

Case	Year	Author	Age	Sex	Complaint	Treatment	Resection	Recurrence
1	2003	Treska V	20	M	Abdominal pain	Laparotomy	Complete	−
2	2013	Nazarewski L	41	F	Asymptomatic	Laparotomy	Complete	−
3	2016	Present case	50	F	Abdominal distention	Laparoscopic surgery	Incomplete	+

There are various clinical symptoms of intra-abdominal lymphangioma. Some patients are asymptomatic whereas others have a palpable mass, abdominal pain, or abdominal distension [8]. Infrequently, patients have an acute abdomen or pan-peritonitis due to infection, perforation, torsion, or rupture of the tumor [9].

Histologically, lymphangiomas are divided into three subtypes: capillary, cavernous, or cystic. However, it was suggested that cystic and cavernous lymphangiomas are not easily distinguishable. In fact, many lymphangiomas have both cystic and cavernous components [10].

The pathogenic mechanism underlying the development of lymphangiomas is unclear. There are several hypotheses for the origination of these tumors, such as abnormal congenital development of the lymphatic system, arising from lymphatic obstructions (such as from infection or surgery), or as a result of local degeneration of lymphatic tissue [11].

Preoperative diagnosis of lymphangiomas is usually difficult with only a few reports in which an accurate preoperative diagnosis was made [12]. Levy et al. described a typical US finding as a well-circumscribed cystic lesion with multiple thin septa and solid echogenicity with a honeycomb pattern coupled with CT findings that indicate either unilocular or multilocular masses with enhancement of the wall and septa by contrast medium [13]. However, in our case, neither multiple septa nor wall enhancement were observed. Because of these atypical findings and the location of the mass, we mistakenly diagnosed the lesion as a simple cyst of the liver.

Histological and immunohistochemical examinations are needed for complete diagnosis of lymphangiomas. At the first operation, the cystic mass originated from the dorsal part of the hepatoduodenal ligament and not from the liver parenchyma. However, this observation alone was not sufficient to make a diagnosis of lymphangioma originating from the hepatoduodenal ligament. Suthiwartnarueput et al. previously reported that immunohistochemical positivity for D2-40, a lymphatic endothelial marker, in the cystic wall endothelium, supported this rare diagnosis [14]. In the present case, endothelium lining the inner-surface of the tumor wall stained positive for D2-40 but negative for markers of mesothelium or vascular endothelial cells, supporting the final diagnosis.

Complete excision is required to avoid recurrence of the cyst and its symptoms if a diagnosis of lymphangioma is made [15, 16]. In this case, we made a diagnosis of cystic lymphangioma after the operation. Close follow-up of the patient verified the recurrence of the disease 6 months after the first incomplete resection. The recurrent cystic mass was removed completely via open laparotomy. Alternative treatments with injections of bleomycin or OK-432 into the cyst may have been effective if complete excision was technically impossible [8].

## Conclusions

Intra-abdominal cystic lymphangiomas, which occur in the hepatoduodenal ligament mimicking a simple liver cyst, are extremely rare, benign tumors. A diagnosis of this tumor requires histological and immunohistochemical support. Complete excision is the treatment of choice to avoid recurrence of the tumor.

## Abbreviations

CT: Computed tomography
HBME-1: Hector Battifora mesothelial epitope-1
US: Ultrasound sonography
